# Pilot Study to Assess Isoflavone Intake in Middle-Aged Italian Subjects

**Published:** 2008-03

**Authors:** Simona Bertoli, Angela Spadafranca, Laura Ravelli, Paola Foti, Daniela Erba, Giulio Testolin, Alberto Battezzati

**Affiliations:** 1 *Department of Food Science, Technology and Microbiology (DISTAM) Agricultural Faculty, University of Milan, Italy;*; 2 *International Center for the Assessment of Nutritional Status (ICANS), University of Milan, Italy*

**Keywords:** daidzein, genistein, isoflavones, Italian diet, soy products

## Abstract

Knowledge of isoflavone (IF) intake in Western populations is scarce, primarily because data about the content of these compounds in non-soy derived foods are incomplete or unavailable. The aims of this study were 1) to enrich the data available in literature about the IF content in traditional Italian foods, 2) to estimate daidzein (D) and genistein (G) intake in an Italian population sample. Eighteen Italian foods have been selected and analysed for IF content by GC-MS; the assessment of IF intake was performed in sixty healthy middle-aged Italian subjects after investigation of their dietary habits by food frequency questionnaire (FFQ). The mean IF intake was 171 ± 261 μg/die (26-1415 μg/die). The mean G intake was greater than D (98 ± 131 μg/die vs 76 ± 131 μg/die). As expected, soy products, even though poorly consumed (27%), resulted the main contributor to IF intake (IF intake was 473.4 ± 440 μg/die vs 75 ± 38 μg/die in soy consumers and non soy consumers respectively *p*<0.001). Among Mediterranean foods, the main contributor resulted fresh bread that is widely consumed (97%). The percentage contribution of the cereal group to mean IF intake was 91%; the legume, fruit and vegetables groups brought a low contribution (3%, 2% and 4% respectively). The total daily IF intake found was low and probably not sufficient to produce biological effects. However more studies are necessary to investigate whether low exposure to IF for a long time could have positive effects on human health.

## INTRODUCTION

Epidemiological studies have shown a lower incidence of chronic degenerative diseases such as hormone-related cancer (1), osteoporosis (2) and cardiovascular pathologies (3) in Asia than in the United States and Europe.

This seems to be related more to environmental factors than to genetic factors: This P *et al* (4) reported that the risk of breast cancer in Asian woman increased after several generations when they emigrated to the United States. Among environmental factors, the typical oriental diet which contains less fat and more fibre than the Western diet seems to play an important role (5). In particular many researchers have proposed the hypothesis that the high consumption of soy in Asia, rich in phytoestrogens such as isoflavones (IF), could be partially responsible for the low incidence of chronic degenerative diseases (6, 7).

Several *in vitro* and *in vivo* studies suggest that IF, specifically daidzein and genistein, could have beneficial effects on human health through different mechanisms, among which estrogenic action (8-10) and antioxidant activity (11, 12).

In a typical Asian diet, the IF intake varies from 20 to 50 mg/day (13). Studies *in vivo* have shown that similar amounts are able to exert biological effects with a positive implication on human health (14-16). On the contrary, soy products are poorly consumed by Western people: this suggests that IF intake is very low, but, until now, few and incomplete data are available, especially regarding the Italian population. From available literature data and the USDA-Iowa State University database, soy and soy-derived products contain relevant amounts of IF and legumes resulted a possible IF source (17). Less characterized are other foods (such as bread, bread substitutes, breakfast cereals and pasta) typical of the Italian diet. The latter foods, although probably poor in IF, could however contribute to Italian IF intake, especially if regularly and/or abundantly eaten. Moreover, we cannot exclude that nowadays some ingredients used for processing some western foods, such as bakery products, are soy derived.

The aims of this pilot study were 1) to analyse the level of daidzein and genistein in some Mediterranean products in order to enrich the data available in literature, and 2) to estimate the level of daidzein and genistein intake in a sample of middle-aged Italian people after investigation of their food habits.

## MATERIALS AND METHODS

### Analysis of daidzein and genistein contents in some Mediterranean products

On the basis of a multicentric study carried out in the years 1994-1996 by INRAN (Istituto nazionale di Ricerca per gli Alimenti e la Nutrizione) (18) to evaluate Italian food consumption, eighteen frequently consumed foods were selected and analysed for IF content.

**Collection and preparation of food samples**. The foods selected belonging to cereal, legume and sweet groups as shown in Table 1, were purchased in local supermarkets (Milan, Italy) or, with regard to bread, in local bakeries (Milan, Italy). In order to minimize sampling error, four samples of each food, cooked when necessary, were pooled, homogenised and freeze-dried. The dried samples were stored at -20°C.

**Table 1:** Daidzein (D) and Genistein (G) contents in selected and analysed foods

	Number of samples	Isoflavone Content								
		Concentration (mg/100g dry weight)				Concentration (mg/100g wet weight)				
		D		G		D		G		D+G
		Mean	sd	Mean	Mean	Mean	sd	Mean	sd	

*Cereal group*										
** Fresh bread,** local bakery	4	13.3	0.1	28.5	1.9	9.5	0	20.2	1.3	29.7
**Fresh oil bread,** local bakery	4	49.1	0.1	45.1	0.9	37.6	0.1	34.6	0.7	72.2
**Fresh soy bread,** local bakery	4	2390	67.6	1752.7	37.4	1806.4	51.1	1324.7	26.2	3131.1
**Fresh wholemeal bread,** local bakery	4	32.6	1.6	39.5	0.8	24.3	1.2	29.4	0.6	53.7
**Pasta. durum wheat,** GS-Milan	4	12.7	1.2	23.7	1	5.0	0.5	9.4	0.4	14.4
**Wholemeal pasta,** Delverde	4	20.8	0.7	34.8	0.9	9.3	0.3	15.6	0.4	24.9
**Crackers,** GS-Milan	4	15.4	0.5	38.1	0.1	14.5	0.5	35.8	0.1	5.3
**Wholemeal crackers,** Bioagricoop	4	6.8	0.9	21.8	4.3	6.6	0.8	21.2	4.2	27.8
*Legume group*										
**Beans (borlotti), tinned,** De Rica	4	7.8	0.2	37.7	0.5	2.2	0.1	10.5	0.1	12.7
**Beans (cannellini), tinned,** De Rica	4	12.3	1.7	44.2	6.7	3.1	10.4	11.1	1.7	14.2
**Chickpeas, Tinned,** GS-Mila	4	13.3	0.7	66.8	1.1	4.3	0.2	21.7	0.4	26.0
**French beans, tinned,** Valfrutta	4	72.5	3.5	77.9	0.5	7.2	0.3	7.8	0.1	15.0
**Lentils, dried,** Select	4	14.4	0.6	28.8	4	12.8	0.6	25.5	3.5	38.3
**Peas, Tinned,** GS- Milan	4	8.4	1	47.5	0.6	1.8	0.2	10.0	0.1	11.8
*Sweets*										
**Dry cookies,** GS-Milan	4	7.7	1.3	31.1	10.8	7.6	1.3	30.7	10.7	38.3
**Soy cookies,** Misura-Colussi	4	2017.5	66.9	2016.6	293.6	1924.7	63.8	1923.8	280.1	3848.5
**Shortbread biscuits,** GS-Milan	4	7.1	0.5	22.0	3.7	6.9	21.5	0.5	3.6	7.4
**Wholemeal cookies,** Vitasystem	4	nd		nd		nd		nd		nd

nd, not detectable

All reagents and chemicals were purchased from Merck (Darmstadt, Germany), except the enzyme cellulase (from Aspergillus Niger) that was purchased from Sigma Chemical (St. Louis, MO, USA). Standards of IF (genistein and daidzein) were obtained from LC Laboratories (Woburn, MA, USA). N-Methyl-N-(t-butyldimethylsilyl) trifluoroacetamide + 1% t-butyldimethylchlorosilane (MTBSTFA + 1% TBDMCS) was obtained from Pierce (Rockford, USA). Deuterated internal standards, genistein-*d_4_* and daidzein-*d_3_*, were kindly provided by Professor K. Wahala, (Department of Clinical Chemistry, Meilhati Hospital, Helsinki University, Helsinki, Finland).

**Quantification of daidzein and genistein in food**. All reagents and chemicals were purchased from Merck (Darmstadt, Germany), except the enzyme cellulase (from Aspergillus Niger) that was purchased from Sigma Chemical (St. Louis, MO, USA). Standards of IF (genistein and daidzein) were obtained from LC Laboratories (Woburn, MA, USA). N-Methyl-N-(t-butyldimethylsilyl) trifluoroacetamide + 1% t-butyldimethylchlorosilane (MTBSTFA + 1% TBDMCS) was obtained from Pierce (Rockford, USA). Deuterated internal standards, genistein-*d_4_* and daidzein-*d_3_*, were kindly provided by Professor K. Wahala, (Department of Clinical Chemistry, Meilhati Hospital, Helsinki University, Helsinki, Finland).

The extraction of daidzein and genistein from food was performed in according to the method described by Liggins (19) with slight modifications. The IF glycosides present in 0.25 g of sample were dissolved in 20 ml of ethanol 80% and homogenated by ultraturrax (model T25 Janke & Kurkel, IKA-Labortechnic). Any insoluble material was filtered off through a filter paper (Whatman no.4 on top of no.1), and the adsorbed IF were washed with fresh ethanol 80%. The filtrate was evaporated at 45°C with rotavapor. 5 ml of 0.1 M acetate buffer, pH5, containing 100 units of cellulase (Aspergillus niger) were added to hydrolyze glycosides to aglycones. Then the samples were extracted three times with 3 ml of ethyl acetate. An internal standard solution (20 μl std daidzein-d3 0.5 μg/ml + 20 μl std genistein-d4 0.5 μg/ml) was added to the organic phase of the extraction and the sample was evaporated under nitrogen.

Quantification of IF was performed by gas chromatography-mass spectrometry (GC-MS), after derivatization of the samples by the addition of MTBSTFA + 1% TBDMCS and acetonitrile (1:1) to form the t-butyldimethylsilyl (TBDMS) derivative, and incubation over-night at room temperature. Analysis of the samples was performed using a gas chromatography interfaced with a single-quadrupole mass spectrometer (Shimadzu, Tokyo, Japan) equipped with a chamber provided with an electron source. Gas chromatography separation was performed on a DB-1MS capillary column, 15 m × 0.25 mm I.D., film thickness 0.25 μm (Agilent Technology, Palo Alto, CA, USA), whose temperature was programmed to rise from 100°C to 300°C in 15 min (30°C/min). The injector temperature was 280°C and the injection volume was 1 μl in SPILT MODE (1:25). Helium (99% purity) was the carrier gas and it flowed at a pressure of 33.2 kPa. The temperature of GC-MS transfer line (interface) was 230°C. The mass spectrometer was operated in electron impact (70eN) mode with positive ions detection and the equipment was set up to perform selective ion monitoring (SIM). The mass spectrometer was adjusted to monitor ions (M-57) at m/z 425 and 428 for unlabeled and labelled daidzein derivatives, and m/z 555 and 559 for unlabeled and labelled genistein derivatives. Identification of IF was achieved by comparing the gas chromatographic retention times and the mass spectra of the food samples with those of authentic standards. In each sample, the quantification of IF was performed using calibration curves obtained injecting derivatized standard solutions at concentrations in the range of 0-40 ng + internal standard solution (20 μl std daidzein-d3 0.5 μg/ml + 20 μl std genistein-d4 0.5 μg/ml). The chromatogram peaks were integrated using the GC-MS data system. The results are expressed as both μg IF/100g wet weight and μg IF/100g dry weight.

### Assessment of daidzein and genistein intake in a sample of middle-aged Italian people

Sixty healthy subjects of Caucasian race and resident in Italy were recruited. The sample was comprised thirty men with mean age of 50 y ± 1.1 (SD) and thirty women with a mean age of 53 y ± 1.1 (SD). Exclusion criteria included diagnosis with a disease or serious medical condition such as cardiovascular pathologies and diabetes, and elite athletes. Approval was obtained by the institutional Ethical Committee of the University of Milan and a consent form was signed by all subjects.

To assess the IF intake in our sample we used data regarding IF food content already available in literature (Table 2) integrated with those obtained by our analyses (Table 1). Moreover, an investigation of the food habits of the subjects was carried out by a food frequency questionnaire processed by INRAN and filled in by a dietician during an interview with each volunteer. The questionnaire was structured in order to evaluate food frequency consumption during 12 months. A list with different servings of common foods was used to assist in checking the estimated amounts of consumed foods.

**Table 2:** Daidzein (D) and Genistein (G) contents of foods not analysed but consumed by the 60 subjects

	Isoflavone Content						
	Concentration (µg/100g dry weight)				Mean concentration (µg/100g wet weight)		
	D		G		D	G	D+G
	Mean	se	Mean	se			

***By Liggins *et al* 2002***							
Breakfast cereals (mean of different kinds)	4.2	na	7.2	na	4.2	7.2	11.4
Rice long grain. white (boiled)	7.9	0.1	11.9	0.6	2.6	3.9	6.5
Broad beans, cooked	nd		nd		nd		
***By Liggins *et al* 2000 (BJN)***							
Asparagus	3.6	1.5	7.9	0.1	0.3	0.6	0.9
Broccoli (Adlercreutz & Mazur 1997)	5.0	na	7.0	na	na	na	
Carrots, raw (Adlercreutz & Mazur 1997)	2.0	na	2.0	na	na	na	
Cucumber, flesh only	nd		6.2	1	nd	0.2	0.2
Aubergine, cooked	5.0	3.7	10.5	2.7	0.3	0.6	0.8
Mushrooms, common. cookeed	nd		18.2	5.4	nd	1.5	1.5
Potatoes, new, cooked	5.5	1.2	14.7	1.3	1.0	2.7	3.8
Pumpkin	26.2	7.9	nd		12.2	nd	12.2
Tomatoes for salad	nd		4.8	3.1	nd	3.3	3.3
Turnip, cooked	9.4	0.9	7.2	7.8	0.7	0.6	1.3
***By Liggins *et al* 2000 (J Nutr Biochem)***							
Apples	nd		nd		nd	nd	
Apricots raw	nd		nd		nd	nd	
Cherries	nd		nd		nd	nd	
Chestnuts	nd		nd		nd	nd	
Clementines	2.7	0.2	27.0	11.9	0.3	2.9	3.2
Figs	2.8	2	7.7	0.3	0.5	1.4	1.9
Melon, canteloupe	nd		4.2	na	nd	0.4	0.4
Oranges, grapefruit	nd		nd		nd	nd	
Peach, raw	nd		nd		nd	nd	
Pears, comice without skin	0.3	na	5.4	1.4	0.0	0.7	0.8
Plums (Victoria), raw	0.5	na	5.5	2.4	0.1	7.4	7.5
Raisins	69.0	2.4	145.8	4	59.0	124.7	183.6
Strawberries	4.5	3	45.7	10.8	0.5	4.6	5.1
Watermelon	nd		nd		nd	nd	
*Dried fruits*							
Apricots dried	5.0	0.3	nd		4.3	nd	4.3
Figs dried	1.9	0.5	4.5	na	1.8	4.2	6.0
Prunes dried	5.2	3.3	10.4	3.5	4.3	8.5	12.8
*Exotic fruits*							
Mango	25.1	4.1	21.2	5.2	3.8	3.2	7.1
Passion fruits	24.5	1.8	40.3	1.1	6.6	10.8	17.4
*Tinned fruits*							
Peaches, tinned in syrup	3.2	na	5.9	na	0.5	1.0	1.5
Pears, tinned in syrup	1.0	0.2	5.2	0.3	0.2	1.0	1.2
Strawberries, tinned in syrup	nd		22.3	10.1	nd	4.0	4.0

Data are from literature as indicated. nd, not detected; na, not available.

## RESULTS

Results regarding the IF content detected in selected foods are shown in Table 1.

All the foods analysed, except wholemeal cookies, showed detectable amounts of IF. As expected, soy products, such as soy cookies and soy bread, resulted the highest source of IF. Cereal products and sweets (soy products excluded) presented an average value of IF higher than the legume group (35.4 ± 20.5 μg/100 g vs 19.7 ± 10.5 μg/100 g wet weight).

**Figure 1:**
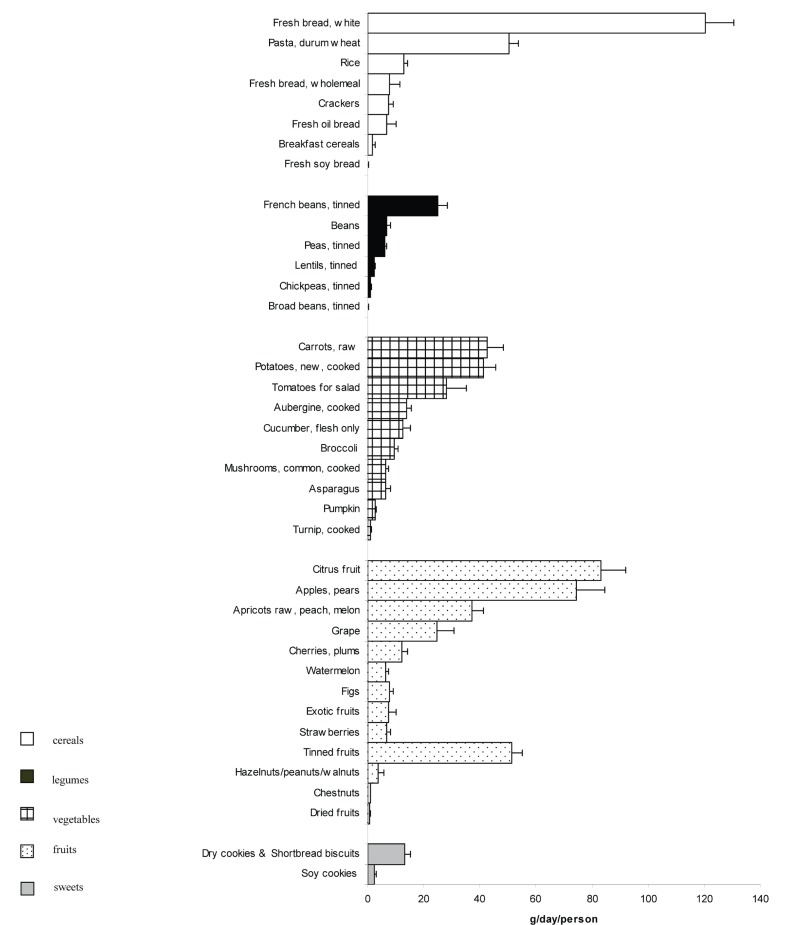
Mean daily intake of cereals, legumes, fruits, vegetables and sweets (n=60).

Figure 1 shows data regarding the mean daily food intake of cereals, legumes, vegetables, fruits and sweets.

The mean IF intake of our sample was 171 ± 261 μg/die, with a very large range (26-1415 μg/die). The mean exposure to genistein results greater than daidzein (98 ± 131 μg/die vs 76 ± 131 μg/die). In soy products consumers, the mean IF intake was 473.4 ± 440 μg/die (range 81.6-1415 μg/die). If we do not consider soy consumers, the IF intake was 75 ± 38 μg/die.

Table 3 shows the percentages of contribution of each food to IF mean daily intake. As expected, soy products, even though consumed only by 27% of the subjects investigated, resulted the main contributor to IF intake assessed. Among typical Mediterranean foods, instead, the main contributor resulted fresh bread that, differently from soy products, was consumed by 97% of subjects. In general, the percentage contribution of the cereals group (sweets included) to mean IF intake was 91 %; the legume, fruit and vegetables groups brought a low contribution (3%, 2% and 4% respectively).

**Table 3:** Food sources of isoflavones (Genistein+Daidzein) and percentage contribution to daily average intake

*Food*	*Contribution %*

Soy cookies	46.2
Fresh bread, white	18.7
Fresh bread, wholemeal	12.2
Fresh soy bread	3.8
Pasta, durum wheat	3.8
Fresh oil bread	2.6
Crackers	2.0
French beans, tinned	2.0
Dry cookies&Shortbread biscuits	1.6
Carrots, raw	0.9
Potatoes, new, cooked	0.8
Tinned fruits	0.6
Beans	0.5
Tomatoes for salad	0.5
Cherries, plums	0.5
Exotic fruits	0.5
Lentils, tinned	0.4
Rice	0.4
Peas, tinned	0.4
Apples, pears	0.3
Hazelnuts/peanuts/walnuts	0.3
Apricot raw, peach, melon	0.3
Strawberries	0.2
Pumpkin	0.2
Chickpeas, tinned	0.1
Breakfast cereals	0.1
Figs	0.1
Aubergine, cooked	0.1
Mushrooms, common, cooked	0.0
Dried fruits	0.0
Asparagus	0.0
Chestnuts	0.0
Cucumber, fresh only	0.0
Turnip, cooked	0.0
Watermelon	0.0
Grape	0.0
Citrus fruit	0.0
Broccoli	0.0
Broad beans, tinned	0.0

## DISCUSSION

The aim of this study was to estimate the level of IF intake in a sample of Italian people after investigation of their food habits using a food frequency questionnaire, and to quantify the IF content in some typical Mediterranean foods.

Dietary IF intake is mainly correlated with the consumption of soy and soy-derived foods (20), however some studies report dietary IF intakes in Western diets, independently of soy products (21). In particular, data available in literature suggest that cereals and legumes, foods widely consumed by Italian people (18), should be considered IF sources (22-25).

The IF content in food may be different on the basis of origin, cultivar and processing. The knowledge of the IF content in cereal and legume products available on the Italian market is limited; thus, any contribution to increase information should be important. In this study in particular, we investigated the IF content of bread, pasta, crackers, legumes and cookies (18), and also considered soy bread and soy cookies.

Our results indicate that the foods analysed are generally a poor source of daidzein and genistein. The richest sources, as expected, are soy products: however the soy products chosen in this study resulted poorer in IF sources than other soy foods, such as soy milk, soy beans, tofu, miso and natto (25-27). The IF content of soy bread and soy cookies could vary depending on the soy flour used. Liggins *et al* (25) showed that the IF content of soy flours could range from not detected to 362 ng/g. This result suggests that different products made with soy flour could have a great variability of IF content.

Soy flour could also be used as ingredient for non soy derived bread. Liggins J *et al* (25) reported levels of daidzein and genistein between 0.1 mg and 10 mg per 100 g wet weight in five different types of bread (brown, granary, wheatgerm, white, wholemeal). Among the breads analysed in our study, white bread was the most commonly consumed by our subjects. Its IF content resulted ten-fold lower than that found by Liggins J (25), fifty-fold lower than that shown by Horn-Ross P *et al* (28) and thirty-fold higher than that reported by Valsta LM *et al* (29). Even if the IF content detected in white bread considered in our study was low, its contribution to total IF daily intake by Italian people could be important due to its high consumption. Turrini A *et al* (18) reported that among cereal products, white bread, followed by pasta, are highly consumed both in terms of mean amounts (respectively 149.5 and 47.1 g) and in terms of percentage of consumers (98.6% and 94.1% respectively). In our pilot study this trend is confirmed because 98.3% of our subjects daily consumed 120 ± 17 g of white bread, and 98.3% consumed 50 g ± 5 g of pasta.

Despite the fact that the mean daily intake of IF evaluated in our subjects was low, it is interesting to note that the group of foods that had a greater influence on the total exposure was cereals (91%), with a relevant contribution of non soy food cereals of 41%. The contribution to the IF intake from soy foods was high (50%), but due more to their high content in IF than to their consumption. In fact, only 27% of our sample habitually consumed soy products and preferably soy cookies, whose mean consumption was only of 10.6 ± 11.8 g/day.

The total daily intake of IF found in Italian people in this study was low and probably not sufficient to produce physiological effects. Epidemiological studies in fact suggest that the minimal amount of IF intake to produce biological effects in human, such as antioxidant and estrogenic activity should be about 40 mg/die (16), a value far from our data. Moreover, we have to specify that the intake of IF determined in our study does not reflect the actual amount to which the subjects were exposed; for this we need to consider the bioavailability of these compounds and it would be interesting to clarify what the efficient levels are and if low exposure to IF carried out for long time could have a positive biological effect.

Our results are in accordance with those of a study carried out in four European countries, Italy included, and that reported IF intakes lower than 1 mg/day. In Italy in particular the estimated mean daily intake was 554 ± 1072 μg/day (17), excluding soy consumers. We found a lower level of IF intake than M.A.J. van Erp-Baart *et al*. (17), probably due to the size of the sample, the age range of the subjects, the method applied for collecting data about dietary habits and the IF database. This study was a pilot study carried out on a small group of subjects with a limited age range (50-60 ys). This age range was considered because phytoestrogens, such as IF, seem to play a role especially in this period of human life (9, 14, 30). Differently, the age range of subjects selected by van Erp-Baart *et al*. (17) was comprised between 18-94 years. Since food habits are related to life style, it is possible that the different age range could have affected IF intake.

In our study to investigate dietary habits we used a food frequency questionnaire (FFQ) instead of the 7 d diary (17, 18, 31). We chose the FFQ in order to have more complete information about food consumption, also including seasonal foods. However, we have to be aware of the fact that the FFQ is a limited method and not very accurate for predicting the actual exposure to a food component *in vivo* (32); a weighed food record would be more suitable. Finally, since the variability of IF contents in food present in literature is high, we thought that the origin of products might have accounted for this. In fact, to our knowledge, this is the first study to report data of IF content of specific Italian products.

In conclusion the present pilot study confirmed that the Mediterranean diet poorly contributes to IF exposure. Among the foods investigated, cereals are the major source, both for their content and for the frequency of consumption by the Italian population, while legumes, fruit and vegetables give a very low contribution. However further studies are necessary to better define IF intake via the Italian diet: for this purpose larger studies are needed, together with a more complete IF database and an accurate monitoring of the change of dietary habits.
